# Dynamic Gesture Recognition with a Terahertz Radar Based on Range Profile Sequences and Doppler Signatures

**DOI:** 10.3390/s18010010

**Published:** 2017-12-21

**Authors:** Zhi Zhou, Zongjie Cao, Yiming Pi

**Affiliations:** School of Electronic Engineering, University of Electronic Science and Technology of China, Chengdu 611731, China; zzhiuestc@163.com (Z.Z.); ympi@uestc.edu.cn (Y.P.)

**Keywords:** terahertz, high-resolution range profile, Doppler, gesture recognition

## Abstract

The frequency of terahertz radar ranges from 0.1 THz to 10 THz, which is higher than that of microwaves. Multi-modal signals, including high-resolution range profile (HRRP) and Doppler signatures, can be acquired by the terahertz radar system. These two kinds of information are commonly used in automatic target recognition; however, dynamic gesture recognition is rarely discussed in the terahertz regime. In this paper, a dynamic gesture recognition system using a terahertz radar is proposed, based on multi-modal signals. The HRRP sequences and Doppler signatures were first achieved from the radar echoes. Considering the electromagnetic scattering characteristics, a feature extraction model is designed using location parameter estimation of scattering centers. Dynamic Time Warping (DTW) extended to multi-modal signals is used to accomplish the classifications. Ten types of gesture signals, collected from a terahertz radar, are applied to validate the analysis and the recognition system. The results of the experiment indicate that the recognition rate reaches more than 91%. This research verifies the potential applications of dynamic gesture recognition using a terahertz radar.

## 1. Introduction

The frequency of terahertz radar ranges from 0.1 THz to 10 THz, which is situated between microwaves and infrared waves [1]. High-resolution range profiles and Doppler signatures can be achieved easily due to the high frequency of terahertz waves. As a result, a terahertz wave has advantages with regards to target detection and recognition [2]. With the development of terahertz radar systems, terahertz imaging [3], and detection [4], there has been much interest in the study of terahertz radar. However, the research on practical applications is rare and gesture recognition using terahertz radar is still an unexplored field.

A great deal of the research into hand-gesture recognition is based on computer vision and contact-based gesture classifications [5]. The performance of vision-based approaches depends strongly on lighting conditions [6]. Contact-based gesture recognition demands individuals to be accustomed with the usage of the interface device, which is not adaptable to new users [7]. As a result, the application of vision-based and contact-based gesture recognition has many limits. In contrast, terahertz radar can not only provide full-time observation of targets [8], but can also work without wearable devices. In addition, terahertz radar can be used for speed and distance detection [9]. It is applicable to the recognition of hand gestures by detecting changes in distance and speed. In recent years, centimeter-wave radar (frequency in the 3–30 GHz range) has been used in gesture recognition systems [10]. However, large-scale motion has usually been studied, which is confined to low resolution. Very recently, a gesture recognition system Soli was designed for some specific usage scenarios based on millimeter-wave radar with a short range (frequency in 60 GHz). Compared with the lower frequency radar system, the terahertz radar has a higher carrier frequency. It’s easy for terahertz radar to achieve wider bandwidth and provide better range resolution, which can precisely capture a change of hand gesture.

Information for tracking, Jet Engine Modulation (JEM), polarization, Doppler shifts, HRRP, and radar images are usually utilized to perform target recognition [11,12,13,14,15]. In this paper, we focus on gesture recognition for terahertz radar using multi-modal signals. Multi-modal signals in terahertz systems include HRRP and Doppler signatures. A range profile of terahertz radar is actually a one-dimensional terahertz radar image. Since terahertz radar has sufficient bandwidth, the shape of the returned wave from a target can easily describe the geometric shape and structure of a target [16]. As a result, a change in targets will definitely lead to a change in range profiles. In addition to the change in the target itself, aspect changes are shown in dynamic gestures. Furthermore, a range profile of a single aspect is sensitive to aspect changes [17]. Therefore, range profiles have been widely used in the target recognition. However, most of the previous studies focused on the target itself. HRRP sequences in continuous time reflect movement characteristics, but gesture recognition is rarely discussed. On the other hand, terahertz radar systems used the theory of Doppler speed detection to measure the offset of a frequency. Doppler signatures obtained from terahertz radar are the velocity information of the target motion, which can be used in the hand-gesture recognition field [18]. Multi-modal signals, including HRRP and Doppler signatures in terahertz radar systems, provide target information for both image and velocity. This property allows terahertz radar signals to carry much more information than single sensors, such as camera, infrared sensors, data gloves, and so on. Since gesture recognition has its advantages, thanks to the characteristics of terahertz waves, gesture recognition represents a promising future development for terahertz radar systems. To our knowledge, there have been no reports regarding gesture recognition in the terahertz region. 

Target classification algorithms include Dynamic Time Warping, Hidden Markov model (HMM), Random Forest, Adaptive Boosting (AdaBoost), and so on [19,20,21,22,23]. The Dynamic Time Warping (DTW) algorithm allows two temporal sequences to be aligned in terms of length, and also allows similarities between them to be measured. Therefore, DTW is frequently used in gesture recognition. In [24], hand gesture data was acquired using a multisensor system and the use of DTW as a fusion processor was studied. In [25], a modified DTW algorithm is designed for gesture recognition using an inertial-sensor based digital pen. In [26], gesture signals acquired from a depth camera were accurately classified using a novel DTW algorithm. In [27], the efficiency of different dynamic time warping methods was compared, based on a database of 2160 simple gestures.

Inspired by the preceding works, gesture recognition using terahertz radar is proposed in this paper. HRRP sequences of hand gestures, in continuous time, are obtained from the received signals of a terahertz radar system. The location parameter features of scattering centers are extracted from HRRP sequences using the Relax algorithm [28]. On the other hand, Doppler signatures are extracted using time-frequency analyses using a terahertz radar. Since two characteristics of hand gestures are obtained, the local distance measure of DTW is extended to deal with vectors at each time point. Furthermore, DTW distance fusion is added to accomplish decision-making. A terahertz radar dataset is built in order to verify the effectiveness of our proposed method. The dataset is composed of 10 different classes of gesture signals performed by five people. The total number of gesture signal samples is 1050. The recognition rates are eventually acquired, based on the dynamic hand gesture dataset. In the experiments, we demonstrate that the recognition scheme proves to be effective in the terahertz region. We also conducted a comparison experiment with project Soli. The experimental results prove the contribution of high resolution of our terahertz radar system and the effectiveness of our method.

The rest of the paper is structured as follows. The terahertz radar system is introduced, and multi-modal signals, including HRRP and Doppler signatures, are achieved in the terahertz region in Section 2. In Section 3, a recognition approach is proposed and a recognition scheme is presented. In Section 4, the experimental data and data preprocessing are discussed. In Section 5, some experimental results are presented. Finally, conclusions are given in Section 6.

## 2. Radar System and Multi-Modal Signals

### 2.1. Radar System

**Figure 1:**
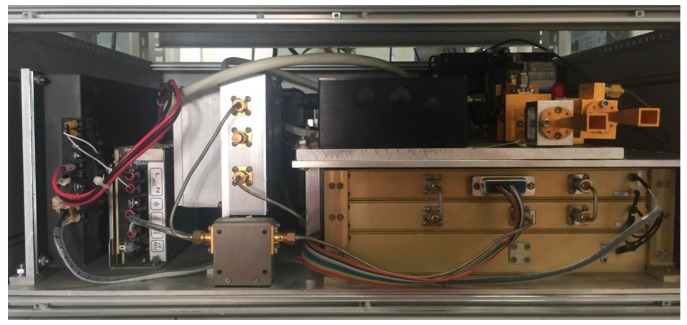
Photograph of the front-end setup.

**Figure 2:**
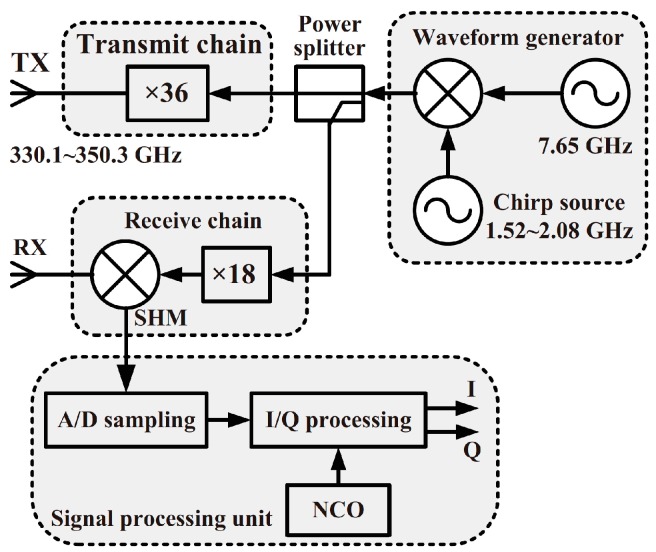
Block diagram of the terahertz radar system. TX is transmitter, RX is receiver, SHM is subharmonic mixer, A/D is Analog-to-Digital, I/Q is In-phase/Quadratic and NCO is numerically controlled oscillator.

The signals of gestures used in this paper were acquired using a terahertz radar. The framework of the terahertz radar comprises four models: wave generator unit, chirp source unit, transceiver unit and signal processing unit. The front-end setup of the terahertz radar is shown in Figure 1. A block diagram of the terahertz radar system is shown in Figure 2. First, a chirp signal (bandwidth: 800 MHz) was generated using the direct digital synthesizer based waveform generator. Then, the chirp frequency was increased from the original 9 GHz to 340 GHz using a transmit (36 multiplication) chain. Downconversion was achieved using a subharmonic mixer pumped by the 165–172-GHz local oscillator signal.

Detailed parameters of the terahertz radar are shown in Table 1.

### 2.2. HRRP Sequences

Considering the linear frequency modulated (LFM) signal we used as the illuminating signal, inverse fast Fourier transform (IFT) was employed to achieve a high-resolution range profile of the target [29].

**Figure 3:**
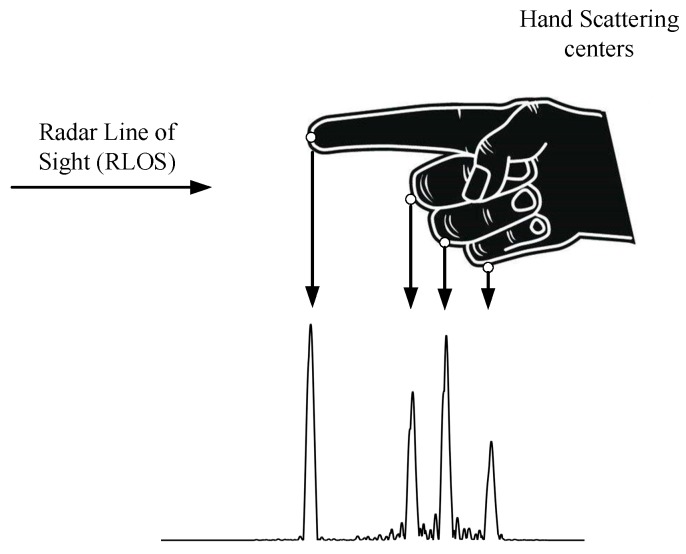
The high-resolution range profile (HRRP) from a hand in the radar line of sight (LOS), in which the circles represent sources of scattering points.

The HRRP of a hand in the radar line of sight is shown in Figure 3. As we can see, the scattering points lie mainly on the fingertips, knuckles, or some other points of the hand.

**Figure 4:**
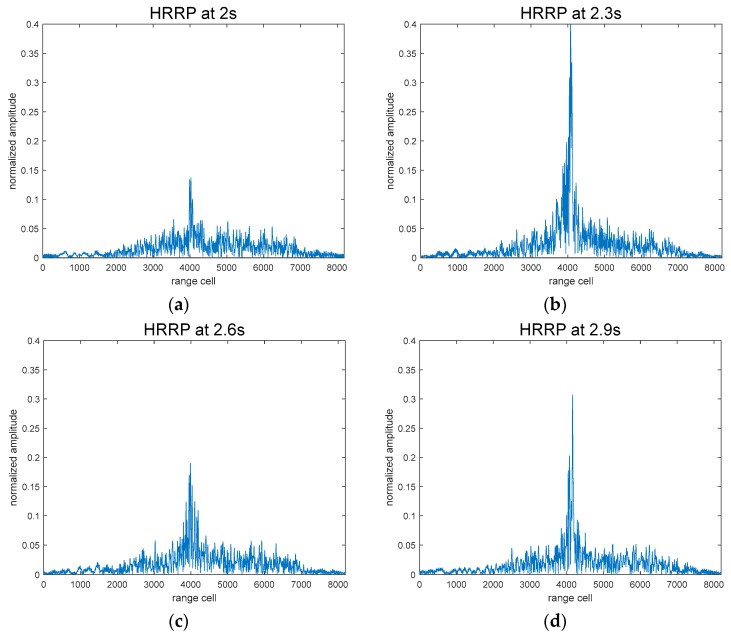

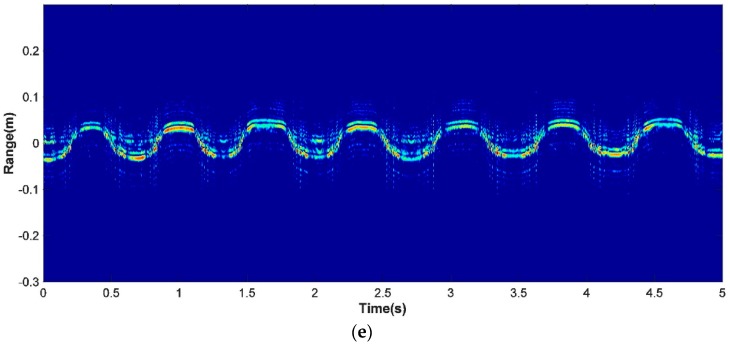
(**a**–**d**) HRRP of a hand at a certain point in time; (**e**) HRRP sequences of a dynamic gesture over five seconds, where the brightness represents the amplitude (**a**–**d**).

As shown in Figure 4, the HRRP sequences in a motion period are a collection of 1D radar images at every sample time [30], which can precisely reflect the motions of gestures. The sampling time in our terahertz system was 1 ms.

### 2.3. Doppler Signatures

Doppler signatures provide information on hand movements, as well as structure for dynamic hand gesture recognition. In the radar line of sight, positive Doppler frequencies will appear when a hand moves in the direction of the terahertz radar. On the other hand, a target moving away from the radar will lead to negative Doppler frequencies [31].

Radar echoes are gained from the terahertz radar and then by FFT (fast Fourier transform) analysis, the time-range profiles are used for Doppler information extraction. Then, the strongest points can be easily determined at each time point. Finally, the method of time-frequency analysis (Short-time Fourier Transform) is employed for the strongest points in continuous time using a sliding Hamming window to extract the Doppler signature from the HRRP sequences [32].

## 3. Gesture Recognition Scheme

### 3.1. Feature Extraction of HRRP Sequences

The HRRP at a given time, with *N* points back scatterings (PBSs), can be represented using a matrix:

$$\left\{\begin{array}{c} f_{1},f_{2},\ldots ,f_{N}\\ \sigma_{1},\sigma_{2},\ldots ,\sigma_{N} \end{array}\right\}$$

The location features of all scattering centers on the target can be represented by a set of parameters $\left\{f_{i},\;i=1,\;2,\;\ldots ,\;N\right\}$. Meanwhile, the amplitude features of all scattering centers of a target can be represented using a set of parameters $\left\{\sigma_{j},j=1,\;2,\;\ldots ,\;N\right\}$. Previous studies have used a variety of methods including alternative projection algorithm (ANPA), method of direct estimation (MODE), estimation signal parameters via rotational invariance techniques (ESPRIT), and the Relax algorithm [33,34,35]. Since the Relax algorithm has the advantages of better robustness variation and convergence speed, it was selected for the extraction of locations and amplitude features in this paper. However, the number of scattering centers from a target relates to the size and electromagnetic scattering characteristics of the target. As a result, the number of scattering centers changes when a dynamic hand gesture is performed. A range profile can be represented by a few of the strongest scattering centers. The features of the *n* (*n* = 5 in this paper) strongest scattering centers were selected to complete the classification.

Supposing that a range profile in $T_{s}$ seconds is acquired, and the length of time required to finish a gesture is T seconds, the number of range profiles during that time would be $M=T/T_{s}$. The parameter-set of HRRP sequences representing a gesture can be described as:

$$\left\{\begin{array}{ccccccc} f_{11} & \sigma_{11} & f_{21} & \sigma_{21} & \cdots & f_{M1} & \sigma_{M1}\\ f_{12} & \sigma_{12} & f_{22} & \sigma_{22} & \cdots & f_{M2} & \sigma_{M2}\\ f_{13} & \sigma_{13} & f_{23} & \sigma_{23} & \cdots & f_{M3} & \sigma_{M3}\\ \vdots & \vdots & \vdots & \vdots & \ddots & \vdots & \vdots \\ f_{1n} & \sigma_{1n} & f_{2n} & \sigma_{2n} & \cdots & f_{Mn} & \sigma_{Mn} \end{array}\right\}$$

In general, the location of the distribution of a target is more robust to variation in the target’s aspect angle than the amplitude [36]; as a result, the locations of the scattering centers were chosen as recognition features of the HRRP sequences:

$$\left\{\begin{array}{cccc} f_{11} & f_{21} & \cdots & f_{M1}\\ f_{12} & f_{22} & \cdots & f_{M2}\\ f_{13} & f_{23} & \cdots & f_{M1}\\ \vdots & \vdots & \ddots & \vdots \\ f_{1n} & f_{2n} & \cdots & f_{Mn} \end{array}\right\}$$

In addition, the computation load is also decreased by 50% by choosing only location features when compared with choosing features related to both location and amplitude. 

### 3.2. Classification Method

DTW is an algorithm that measures the similarity between two sequences (mostly in different lengths). It aims to find the shortest path by twisting and bending a time series. Therefore, the DTW algorithm is widely used in the fields of voice, handwriting, and gesture recognition. This feature of the DTW algorithm also makes it applicable to gesture recognition, based on terahertz radar.

In the classic DTW algorithm, a local distance measure is defined to acquire the similarity between a training sample and a number of testing samples by finding an optimal path. Suppose we have a test template named *s* of length *I* and a training template named *t* of length *J*, non-linear matching is achieved between the two templates by finding an optimal path using an *I*-by-*J* matrix. The distance function, denoted as $d_{f}\left(i,j\right)$, is the Euclidean distance, typically used for similarity measures. A warping path, *D*, can be described as:

(1) $$D=\left\{d\left(i\left(q\right),j\left(q\right)\right)|\begin{array}{l} q=1,\ldots ,Q,\\ \max\left(I,J\right)\leq Q\leq I+J-1 \end{array}\right\}$$

There are several constraints to be discussed in terms of path selection.

Bounded constraints: The starting point is fixed at (1, 1) and point (*I*, *J*) is always the end point. It is possible that the gestures may take different times to complete, but the order of the motion cannot be changed.

Continuity constraints: no breaks in the path are guaranteed using continuity constraints.

Monotonicity constraints: the monotonicity constraints require the character of a gesture signal to be clustered in monotonically increasing order.

Slope constraints: the slope constraints ensure that there is no excessive sway in the path. The slope is usually restricted to (0.5, 2).

Based on the constraints above, the similarity measure, DTW(t,s), between the training template and the testing template can be acquired from the accumulated local distance by following path *D*. The minimal overall distance of the warping path is the best alignment between the testing sample and the training sample:

(2) $$DTW\left(\vec{t},\vec{s}\right)=\underset{D}{\arg\min}\left(\frac{\sum_{q=1}^{Q}d\left(i\left(q\right),j\left(q\right)\right)}{k}\right)$$

where *k* is used for normalization.

The classic *DTW* method has mainly been applied to find the optimal path between one-dimensional temporal sequences. However, the location features of HRRP sequences and Doppler signatures are not one-dimensional temporal sequences, due to the high-resolution capabilities of terahertz radiation. On the other hand, the extensions to multi-feature temporal sequences are straightforward. Thus, the classic *DTW* algorithm needs to be improved.

In the classic *DTW* algorithm, the difference in only two values ($T_{i}$ and $S_{j}$) is calculated as a similarity measure. To extend to the gesture recognition using a terahertz radar, the difference of two vectors ($T_{i}^{N}$ and $S_{j}^{N}$) is denoted as a similarity measure instead of the difference between two points. We focused on two vector similarity measurements (Euclidian distance and the Cosine correlation coefficient) in this paper. The performances of the two kinds of local distance measures is discussed in the next section. Considering the characteristics of HRRP and Doppler signatures, the Euclidian distance is defined as:

(3) $$d_{E}\left(T_{i}^{N},S_{j}^{N}\right)=\sqrt{\sum_{n=1}^{N}{\left(T_{i\left(n\right)}-S_{j\left(n\right)}\right)}^{2}}$$

The Cosine correlation coefficient can be described as:

(4) $$d_{C}\left(T_{i}^{N},S_{j}^{N}\right)=1-\frac{\sum_{n=1}^{N}S_{j\left(n\right)}T_{i\left(n\right)}}{\sqrt{\sum_{n=1}^{N}S_{j\left(n\right)}^{2}}\sqrt{\sum_{n=1}^{N}T_{i\left(n\right)}^{2}}}$$

**Figure 5:**
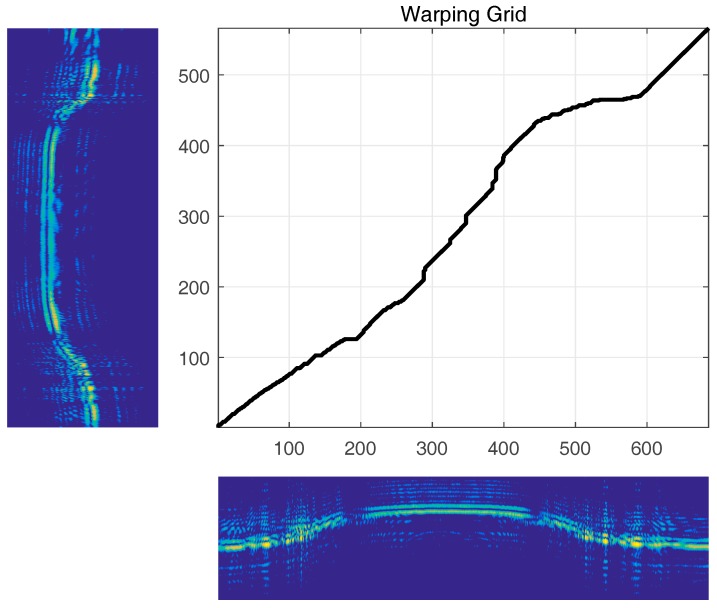
Warping grid using Euclidian distance.

A test template was compared using one of the training templates by seeking the optimal path between the two templates. The optimal path of the two templates is illustrated in Figure 5. By convenience, the shorter template is aligned along the Y-axis.

Since we had two types of signals, HRRP sequences and Doppler signatures, it was necessary to take data fusion into account. The training templates, D(M×I), and test templates, F(M×J), represent location features of HRRP sequences. The training templates, G(O×K), and test templates, H(O×L), represented Doppler signature sequences. Thus, the overall Euclidian distance after data fusion was defined as:

(5) $$DTW_{e}=W\left(1\right)\times DTW_{e}\left(D,F\right)+W\left(2\right)\times DTW_{e}\left(G,H\right)$$

The overall Cosine correlation coefficient is defined as:

(6) $$DTW_{c}=V\left(1\right)\times DTW_{c}\left(D,F\right)+V\left(2\right)\times DTW_{c}\left(G,H\right)$$

where elements *W* and *V* are positive. Weight vectors *W* and *V* were used to accomplish the data fusion and enhance the accuracy of hand gesture recognition. When all the elements of the vectors are equal, the normal Euclidian distance and Cosine correlation coefficient could be obtained.

### 3.3. Recognition Scheme Overview

**Figure 6:**
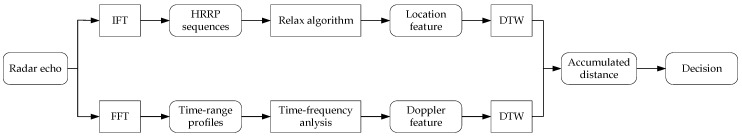
Overview of the recognition system.

The recognition system was primarily composed of data preparation and DTW fusion. Data preparation mainly consisted of the acquisition of HRRP sequences and Doppler signatures. A threshold value filter and amplitude normalization were also included. A decision was made after counting the accumulated distance. The framework of the recognition system is shown in Figure 6.

## 4. Experimental Data

**Figure 7:**
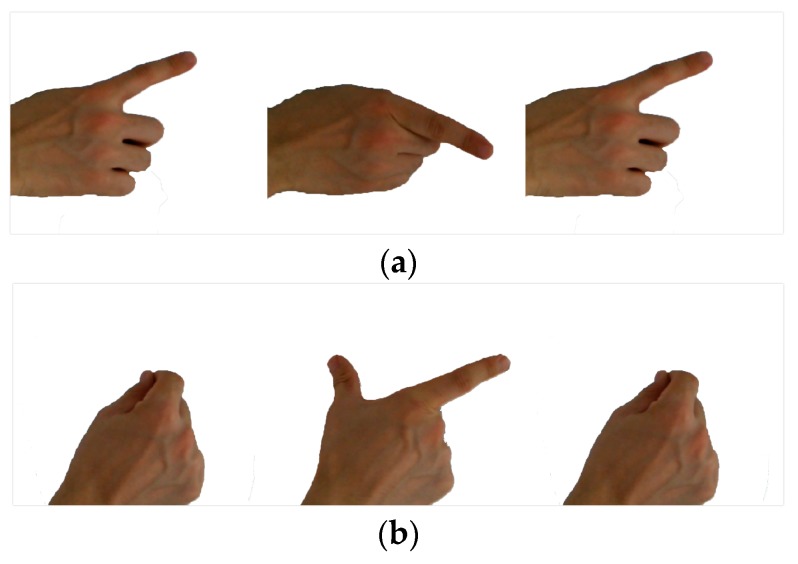

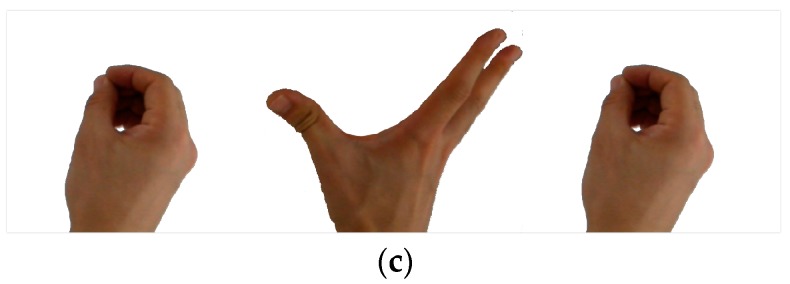
Start, middle, and end point of a gesture: (**a**) G1: Swing the index finger so that it is perpendicular to the radar line-of sight, from left to right, and then from right to left; (**b**) G2: Keep fingers in the direction of the LOS, pinch the thumb and index finger; (**c**) G3: Keep fingers in the direction of the LOS, pinch all fingers together and then spread them out, then pinch all the fingers together again.

Ten types of gestures were selected to acquire the experimental data set in this work. Three of the gestures are shown in Figure 7. Due to different behavioral habits, an action may not be the same when different people make the same gesture. Furthermore, when one person makes a gesture at a different time or under different circumstances, there are still differences that are mainly displayed in terms of range and speed of motion. Therefore, five individuals were chosen to complete ten gestures in order to verify the effectiveness and robustness of the recognition scheme.

**Figure 8:**
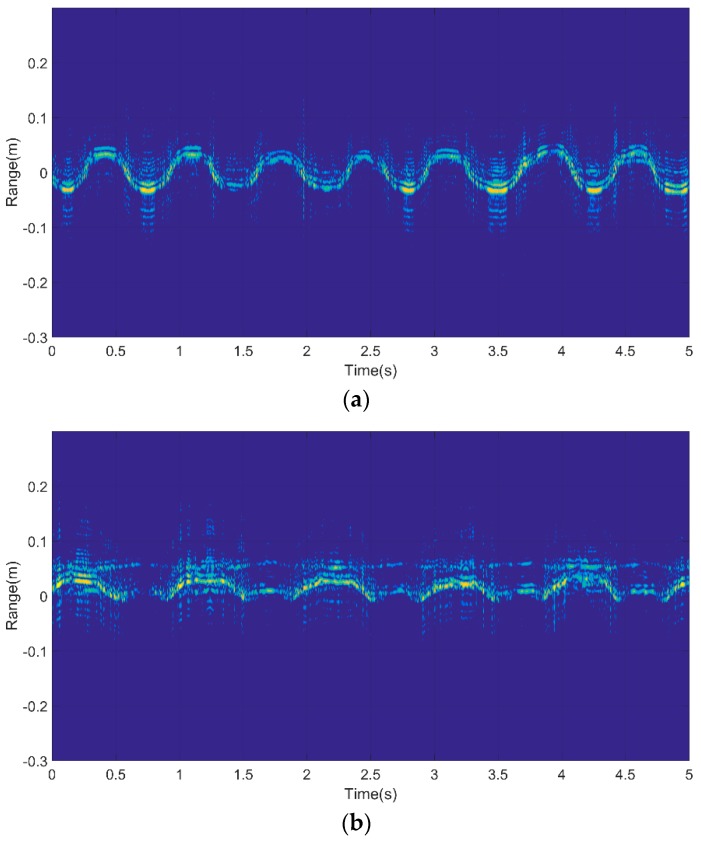

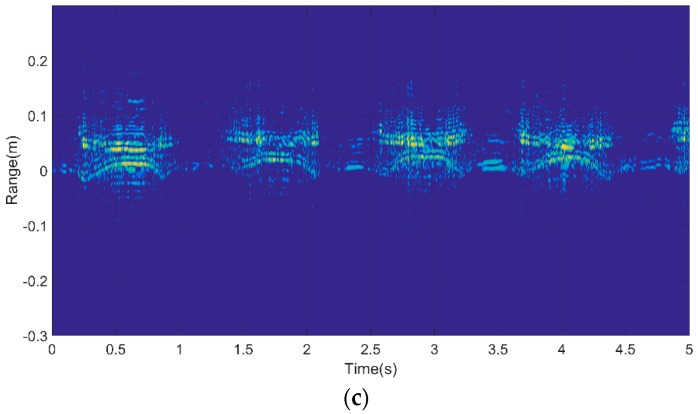
HRRP sequences of three gestures: (**a**) G1; (**b**) G2; (**c**) G3.

The HRRP sequences of the gestures used in this paper were acquired using a Linear Frequency Modulated (LFM) signal. Corresponding to the three gestures shown above, HRRP sequences are shown in Figure 7. In each of the images, several periods of continuous gesture are shown; they span five seconds, and were collected from the same person. As we can see from Figure 8a, fingers started at a position nearest to the radar receiver, then moved to the furthest point from the radar receiver, and finally returned to the starting point. The HRRP sequences reflect the structure and movement of the hand.

**Figure 9:**
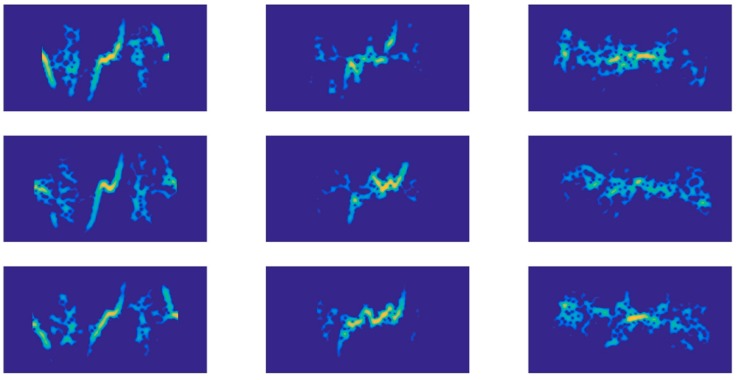
Doppler signatures of three hand gestures for three samples. Three pictures in each column represent G1, G2 and G3, respectively.

The Doppler signatures of the three gestures are illustrated in Figure 9. It is shown that the same gestures had similar Doppler signatures (similarity across each column), and different gestures had distinct Doppler signatures (differences among each row). However, the length of the Doppler signatures may be different. The bright area concentrated around zero frequency meant the static part of the hand, which contributed nothing to recognition. Therefore, $P_{ij}=\min\left(P\right)$ was set around the zero frequency to remove the static part from our recognition scheme.

## 5. Experimental Data Preprocessing

It is inevitable that an individual will perform several types of gestures constantly. However, there is a short-time pause, or a motion speed closing on zero, when a previous action was performed. Therefore, the total time spent on a single gesture could be determined using near-zero velocity detection in HRRP sequences.

**Figure 10:**
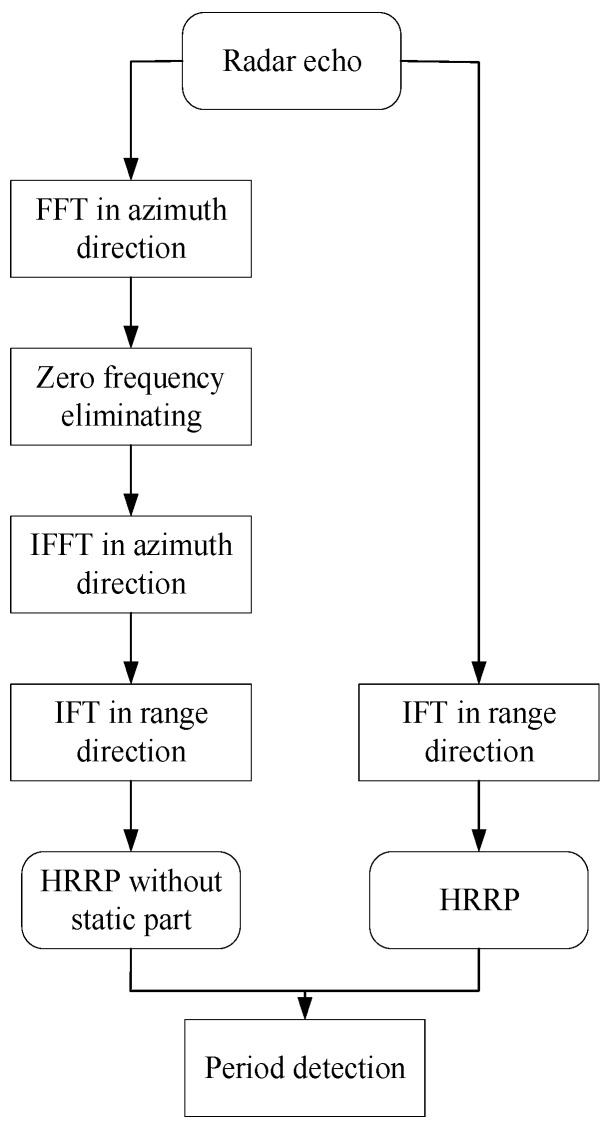
Gesture period acquisition.

**Figure 11:**
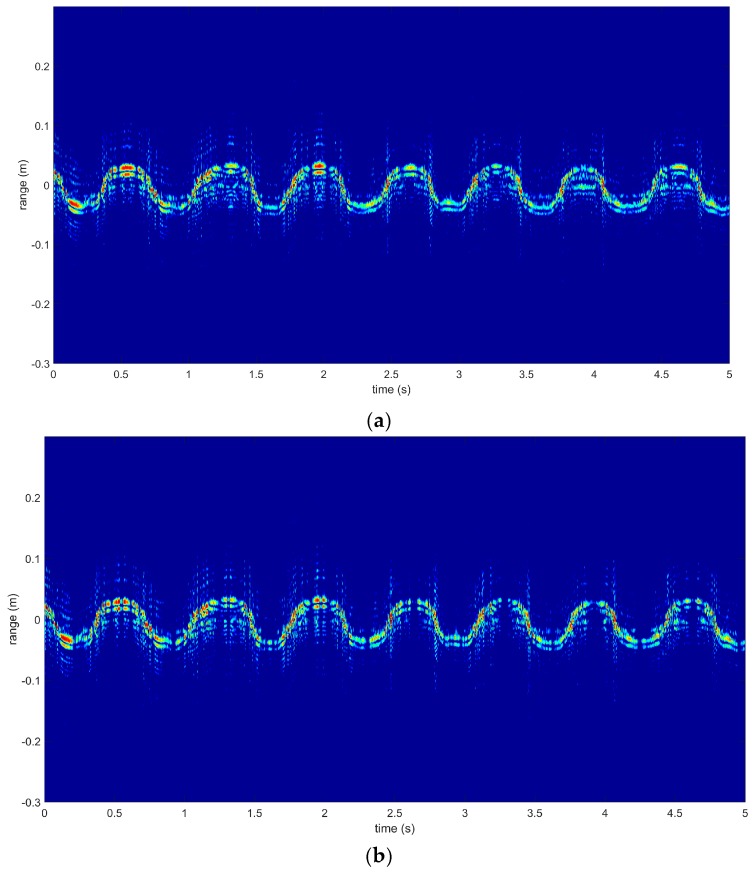

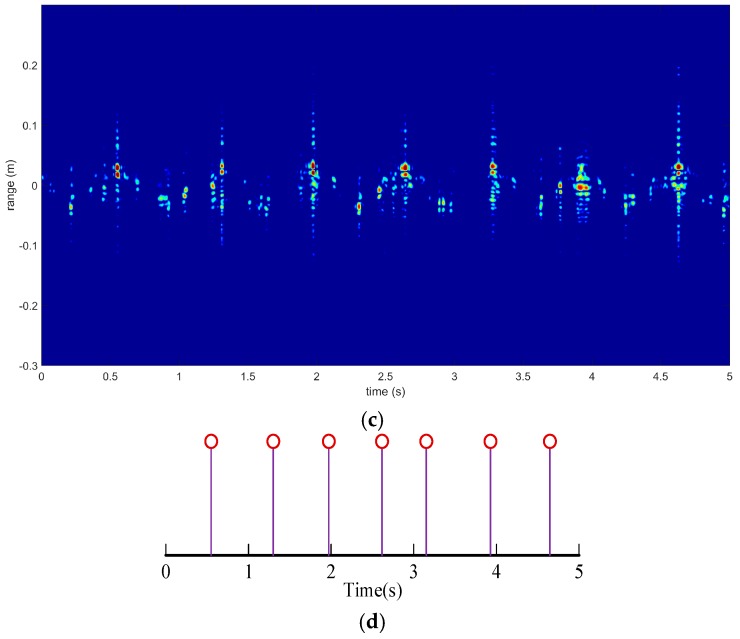
(**a**) HRRP sequences over five seconds; (**b**) HRRP sequences without the static part; (**c**) static part; and (**d**) time points of finished gestures.

Considering the properties of LFM radar signals, we could translate radar echoes from the time domain to the frequency domain using FFT in the azimuth direction. The static parts were actually the zero frequency points in the frequency domain, according to the Doppler theory. The signal was translated back to the time domain after removing the zero frequency points in the frequency domain. Then, continuous HRRP sequences, without the static part, could be acquired using IFT in the range direction. The target is still when the HRRP of a target is totally removed after eliminating the zero frequency. The flowchart for period determination is shown in Figure 10. The period acquisition, based on HRRP sequences, is shown in Figure 11. Moreover, the static part in HRRP sequences had a direct influence on the accuracy rates of recognition. Therefore, data preprocessing can also improve the recognition rates.

## 6. Experimental Results

In this experiment, there were ten types of gestures used to analyze the effectiveness of the recognition system. To verify our system, five individuals were selected to make ten gestures. Each person repeated the same gesture 21 times. The total number of gesture signal samples was 1050. Five samples of each gesture were chosen to be the training samples. The total number of training samples was 50 and the remaining 1000 were set as test samples in the experiments. 

The recognition rate of a single feature using the Cosine correlation coefficient is show in Table 2.

The recognition rates with Euclidian distance and Cosine correlation coefficient are shown in Table 3 and Table 4, respectively.

HRRP sequences of continuous time can precisely capture changes by the target, as well as the movement characteristics. The Doppler feature can, not only reflect movement, but can also eliminate the influence of the static and useless part of the hand. The two signatures can be viewed as complementary features in the recognition system. As we can see from Table 2, the recognition rate of HRRP was 88.2%, and the recognition rate of the Doppler feature was 89.2%. The total recognition rate of using both HRRP and the Doppler feature was 91.5%, which is better than that of a single feature. The experimental results prove that both HRRP and Doppler provide useful information. A classification method based on both HRRP and Doppler has a lot of potential. The recognition system has been proved to be effective using both HRRP sequences and Doppler signatures. The recognition rate with the Cosine correlation coefficient (91.5%) was higher than that of the Euclidian distance (89.3%), which means that the Cosine correlation coefficient was a more effective measure in our recognition scheme. The total classification rate can reach 91.5%. The experimental results indicate that our recognition system, in the terahertz regime, has the ability to achieve a good performance.

**Figure 12:**
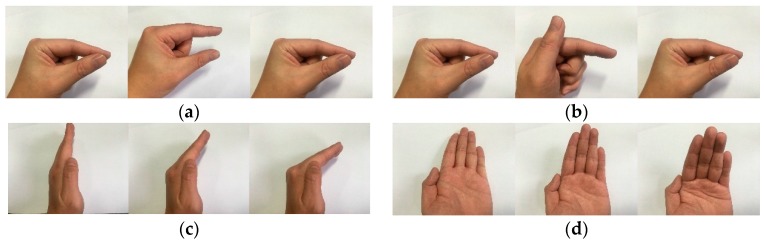
Start, middle, and end points of four gestures. (**a**) Virtual Button; (**b**) Virtual Slider; (**c**) Horizonal Swipe; (**d**) Vertical Swipe.

We built another dataset of four gestures, according to the Soli Project [37], as shown in Figure 12.

Five individuals are selected to make the four gestures. Each one repeats the same gesture for 100 times at various locations within a 10-m range of the sensor (compared with the 30-cm range of Soli). The total number of gesture samples is 2000. 250 samples of each gesture are chosen to be the training samples. The total number of training samples is 1000 and the remaining 1000 ones are set as the test samples in the experiment. The recognition rate of Soli project and our system is shown in Table 5.

This experimental result proves the contribution of the high resolution of our terahertz radar system, as well as the effectiveness of our method.

## 7. Conclusions

A novel system for hand-gesture recognition in the terahertz regime was proposed in this paper. In our recognition system, multi-modal signals, including HRRP sequences and Doppler signatures, were used for gesture recognition. A benchmark data set was created to evaluate the recognition system. The DTW algorithm was improved and was applied to the classification system in the terahertz regime. We have demonstrated the effectiveness of this recognition system.

## Figures and Tables

**Table 1:** Terahertz radar parameters.

Parameters	Value
Carrier frequency	340 GHz
Bandwidth	28.8 GHz
Sampling frequency	1.5625 MHz
Pulse repetition frequency	1000 Hz
Cover Range	10 m
Range Resolution	5 mm

**Table 2:** Recognition rate of a single feature using the Cosine correlation coefficient distance.

Type	G1	G2	G3	G4	G5	G6	G7	G8	G9	G10	Average
HRRP	97	83	81	91	87	82	89	92	90	90	88.2
Doppler	95	89	82	88	88	86	92	92	91	89	89.2

**Table 3:** Recognition rate with Euclidean distance.

Type	G1	G2	G3	G4	G5	G6	G7	G8	G9	G10
G1	97	0	1	0	0	0	1	0	0	1
G2	0	84	5	0	0	1	1	2	0	7
G3	0	4	82	0	3	2	1	3	1	4
G4	0	0	1	93	1	1	0	0	1	3
G5	0	5	1	0	86	3	5	0	0	0
G6	0	0	1	7	4	83	0	3	0	2
G7	0	0	0	3	0	0	95	1	1	0
G8	0	2	2	0	0	0	0	93	0	3
G9	0	1	0	5	0	0	0	1	93	0
G10	4	0	0	0	3	0	5	0	1	87

**Table 4:** Recognition rate with Cosine correlation coefficient distance.

Type	G1	G2	G3	G4	G5	G6	G7	G8	G9	G10
G1	98	0	1	0	0	0	1	0	0	0
G2	0	90	0	1	3	0	0	1	1	4
G3	2	1	87	0	2	6	0	1	1	0
G4	2	0	0	92	1	0	1	0	1	3
G5	0	4	1	0	91	0	2	0	1	1
G6	1	1	1	0	1	87	1	2	1	5
G7	0	1	0	2	0	1	94	0	0	2
G8	0	0	2	2	0	2	1	93	0	0
G9	0	0	0	1	3	0	0	3	92	1
G10	5	0	1	0	0	1	2	0	0	91

**Table 5:** Recognition rate comparison.

	Soli Project	Our System
Recognition rate (%)	92.10	96.70
